# Toxicant Disruption of Immune Defenses: Potential Implications for Fetal Membranes and Pregnancy

**DOI:** 10.3389/fphys.2020.00565

**Published:** 2020-05-29

**Authors:** Sean M. Harris, Erica Boldenow, Steven E. Domino, Rita Loch-Caruso

**Affiliations:** ^1^Department of Environmental Health Sciences, School of Public Health, University of Michigan, Ann Arbor, MI, United States; ^2^Department of Biology, Calvin College, Grand Rapids, MI, United States; ^3^Department of Obstetrics and Gynecology, University of Michigan Medical School, Ann Arbor, MI, United States

**Keywords:** fetal membranes, toxicant pathogen interactions, preterm birth (PTB), pregnancy, trichloroethylene (TCE)

## Abstract

In addition to providing a physical compartment for gestation, the fetal membranes (FM) are an active immunological barrier that provides defense against pathogenic microorganisms that ascend the gravid reproductive tract. Pathogenic infection of the gestational tissues (FM and placenta) is a leading known cause of preterm birth (PTB). Some environmental toxicants decrease the capacity for organisms to mount an immune defense against pathogens. For example, the immunosuppressive effects of the widespread environmental contaminant trichloroethylene (TCE) are documented for lung infection with *Streptococcus zooepidemicus*. Group B *Streptococcus* (GBS; *Streptococcus agalactiae*) is a bacterial pathogen that is frequently found in the female reproductive tract and can colonize the FM in pregnant women. Work in our laboratory has demonstrated that a bioactive TCE metabolite, S-(1, 2-dichlorovinyl)-L-cysteine (DCVC), potently inhibits innate immune responses to GBS in human FM in culture. Despite these provocative findings, little is known about how DCVC and other toxicants modify the risk for pathogenic infection of FM. Infection of the gestational tissues (FM and placenta) is a leading known cause of PTB, therefore toxicant compromise of FM ability to fight off infectious microorganisms could significantly contribute to PTB risk. This Perspective provides the current status of understanding of toxicant-pathogen interactions in FM, highlighting knowledge gaps, challenges, and opportunities for research that can advance protections for maternal and fetal health.

## Introduction

Preterm birth (PTB), or birth <37 weeks gestation, is a significant health problem with lasting consequences. Preterm birth affects more than 1 in 10 babies in the United States as well as globally (March of Dimes et al., 2012; Martin et al., 2019). Babies born preterm are at increased risk for numerous adverse health outcomes later in life, including neurological (Allin et al., 2006), lung (Pike and Lucas, 2015), and intestinal issues (Behrman et al., 2007). In a recent study, Grosse et al. (2017) estimated total medical costs associated with PTB in the United States to be between $6 and 14 billion per year. The fetal membranes (FM), which surround and protect the fetus during pregnancy, play a critical role in both term and preterm labor. In addition to providing a physical barrier, the FM are an important line of defense against pathogenic microorganisms that ascend the reproductive tract (Romero et al., 2007). Notably, pathogenic infection of the gestational tissues (FM and placenta) is a leading known cause of PTB (Goldenberg et al., 2000, 2008).

Epidemiology studies have identified a diverse array of factors associated with PTB (Goldenberg et al., 2008; Ferguson et al., 2013, 2019; Torchin and Ancel, 2016; Vogel et al., 2018). These include exposure to a range of toxic substances including air pollution (Liu et al., 2019), cigarette smoke (Soneji and Beltran-Sanchez, 2019), polyfluoroalkyl substances (Sagiv et al., 2018), polybrominated diphenyl ethers (PBDEs; Peltier et al., 2015), phthalate esters (Ferguson et al., 2014), lead (Taylor et al., 2015), and arsenic (Ahmad et al., 2001). Additional factors include infection with pathogenic bacteria (Bianchi-Jassir et al., 2017) and exposure to high outdoor air temperatures (Zhong et al., 2018; Gronlund et al., 2020). On a mechanistic basis, these exposures are thought to act by triggering oxidative stress and/or inflammatory pathways that are part of the normal labor process of weakening the membranes (Menon et al., 2011; Romero et al., 2014; Wallace et al., 2016; Ha et al., 2018). While biologically plausible, these mechanisms remain poorly understood.

Despite known examples of toxicant-induced immunosuppression occurring in organs such as the lung (Aranyi et al., 1986; Mitchell et al., 2009; Selgrade and Gilmour, 2010), toxicant mediation of immune responses to bacterial infection in FM is largely unexplored. This review focuses on our current understanding about environmental toxicants, pathogenic bacteria and interactions between the two in FM. Due to the potential lifelong health impacts of PTB (Allin et al., 2006; Behrman et al., 2007; Pike and Lucas, 2015) and the critical role that the membranes play in healthy pregnancy (Menon and Moore, 2020), a deeper understanding of these interactions has significant public health implications.

## Anatomy and Function of the Fetal Membranes

The FM are a heterogenous tissue with multiple cell types that make up two distinct layers, the inner amnion (surrounding the fetus) and the outer chorion (Strauss, 2013). The amnion layer is composed of a single amnion epithelial cell layer and dense layer of collagen fibrils synthesized by fibroblasts (Verbruggen et al., 2017). The chorion is composed of trophoblasts that are in close contact with maternally derived decidual cells (Wang et al., 2018; Menon and Moore, 2020). The FM also include a small number of resident innate immune cells (macrophages and monocytes) (Osman et al., 2003).

The culmination of a healthy pregnancy is marked with increased prostaglandin secretion, activation of matrix metalloproteinases, and recruitment of immune cells, leading to myometrial contractions, rupture of the membranes, and cervical ripening, respectively (Vadillo-Ortega et al., 1996; Hernandez-Guerrero et al., 2000; Challis et al., 2009; Yellon, 2019). Although our understanding of the role of FM in the initiation of labor remains incomplete, it is widely accepted that they contribute to the parturition pathway. As pregnancy progresses, the FM secrete increasingly more cytokines and chemokines, which leads to prostaglandin synthesis and release (Mesiano, 2007; Kota et al., 2013) as well as immune cell recruitment in the gestational compartment (Osman et al., 2003). Because the FM abut the uterine muscle (myometrium), they are important as a source of prostaglandins that stimulate uterine contractions in labor.

Furthermore, in normal pregnancies, FM undergo a process of weakening leading up to rupture soon after the start of uterine contractions (Menon, 2016; Menon et al., 2016). Molecular signaling pathways, such as oxidative stress and inflammation, as well as mechanical forces contribute to the weakening of the membranes near term, a process characterized by cellular senescence and aging of the membranes (Menon et al., 2016). Rupture usually occurs in a structurally weak region of the membranes with a thinner chorion that overlies the cervix, referred to as the zone of altered morphology (ZAM; McLaren et al., 1999; McParland et al., 2003; Marcellin et al., 2017).

Premature rupture of the FM, or PROM, is characterized by rupture of the FM more than one hour before the onset of labor. PROM occurring after 37 weeks of pregnancy typically presents relatively few complications. However, pPROM, or preterm premature rupture of the FM (i.e., PROM that occurs prior to 37 weeks of gestation) is associated with severe adverse pregnancy outcomes and is frequently associated with asymptomatic intrauterine infection (Mercer, 2004; Caughey et al., 2008; Huang et al., 2018). Examples of associated adverse neonatal outcomes include respiratory distress syndrome, pulmonary hypoplasia (Nourse and Steer, 1997; Linehan et al., 2016), and neurological outcomes (Manuck and Varner, 2014). pPROM affects around 1–3% of pregnancies (Huang et al., 2018).

## Fetal Membranes as a Target of Bacterial Pathogens

Intrauterine bacterial infection is well established as a cause of PTB (Romero et al., 2014). It is estimated that intrauterine infection accounts for at least 25–40% of PTBs (Goldenberg et al., 2008). Both placenta and FM from preterm and pPROM pregnancies have been shown to be more likely to contain bacterial DNA and a higher level of diversity in bacterial species compared to term pregnancies (Jones et al., 2009). Pathogenic bacteria associated with pPROM and PTB include species from genera such as *Staphylococcus*, *Escherichia, Mycoplasma, Ureaplasma*, and *Streptococcus* (Larsen and Hwang, 2010; Oh et al., 2010; Fortner et al., 2014; Zeng et al., 2014; Kong et al., 2019).

The predominant mechanism by which bacteria enter the gestational compartment causing intrauterine infection is through the ascending pathway by which bacteria first colonize the vagina and cervix, migrate to and then cross the FM, and then colonize the amniotic cavity and fetus (Goldenberg et al., 2000). Therefore, the FM play a critical role as a barrier to bacterial entry.

In addition to providing a physical barrier to protect against infection, the FM provide crucial immunological defense against pathogenic microorganisms that ascend the reproductive tract. The FM actively secrete antimicrobial peptides, such as human beta defensins, lactoferrin, and cathelicidin, to inhibit bacterial infection (Kjaergaard et al., 1999; King et al., 2007a, b; Boldenow et al., 2013). Furthermore, the choriodecidual cells as well as resident innate immune cells are capable of secreting proinflammatory cytokines such as IL-1β, IL-6, IL-8, and TNF-α, which help signal for additional immune cell recruitment (Challis et al., 2009; Yockey and Iwasaki, 2018). Proinflammatory cytokines can also potentially trigger increased release of prostaglandins and proteases, which are key molecular triggers of parturition (Norwitz et al., 1992; Mitchell et al., 1993; Brown et al., 1998; Young et al., 2002; Myatt and Sun, 2010; Romero et al., 2014). Even when bacteria do not infect the amniotic compartment, these proinflammatory responses to bacterial infection in the FM can lead to adverse pregnancy and neonatal outcomes (Adams Waldorf et al., 2011; Burd et al., 2012; Garcia-Flores et al., 2018).

Much of what is currently known about toxicant-bacteria interactions in FM comes from experiments using either *Streptococcus agalactiae*, commonly known as Group B *Streptococcus* (GBS). Group B *Streptococcus* infection in pregnant women is the leading cause of infectious neonatal morbidity and mortality in the United States (Verani et al., 2010). Group B *Streptococcus* induces preterm labor in non-human primates (Gravett et al., 1996; Boldenow et al., 2016). In women, GBS infection is associated with PTB at less than 32 weeks gestation (Hillier et al., 1991) and with chorioamnionitis, an inflammation of the chorion layer of the FM (Anderson et al., 2007). A recent publication from our laboratory showed that GBS inoculation caused a release of molecular effectors of parturition (matrix metalloproteinases and prostaglandin E2) from human FM explant punches *in vitro* (Park et al., 2018). In addition, pathway analysis of transcriptomic responses showed that pathways related to inflammation and PTB were activated by GBS inoculation (Park et al., 2018). Studies from our laboratory showed that a metabolite of trichloroethylene (TCE), a common environmental contaminant, modifies innate immune response to GBS in FM explants (Boldenow et al., 2015). Other groups have shown similar effects with other toxicant-bacteria combinations (e.g., carbon monoxide and *Escherichia coli* (Klimova et al., 2013). Although rarely explored, interactive effects between pathogens and toxicants in gestational tissues are plausible and have significant implications for maternal and fetal health.

## Fetal Membranes as a Target of Environmental Toxicants

Pregnant women are exposed to a multitude of diverse environmental contaminants through drinking water, food packaging, air pollution workplace exposures, and other sources (Mitro et al., 2015). Ubiquitous environmental contaminants such as lead, cadmium, PBDEs, bisphenol A, and phthalates have been detected in human FM (Miller et al., 2009; Kot et al., 2019) and amniotic fluid (Miller et al., 2012; Geer et al., 2015), demonstrating that contaminants can come into contact with the FM either through blood flow to the decidua or via the amniotic fluid. Numerous epidemiology studies have found associations between exposures to environmental toxicants and increased risk of pPROM. These include toxic substances such as lead (Huang et al., 2018), ambient air pollution (Wang et al., 2019) and cigarette smoke (England et al., 2013). These epidemiology studies along with the detection of toxicants in human FM support the role of FM as a target of toxicant effects related to adverse pregnancy outcomes.

### Toxicants Activate Pathways Involved in Fetal Membrane Rupture and PTB

Consideration of the FM as a mediator of toxicant effects is plausible based on their important role in membrane rupture and in the initiation of labor. As recently reviewed by Menon (2016), Menon et al. (2019), and Menon and Moore (2020), the FM contribute to the activation of labor and membrane rupture through a variety of molecular signaling pathways involving hormones, inflammatory cytokines, phosphorylated MAPK p38, reactive oxygen species and prostaglandins. Pro-inflammatory cytokines such as IL-1β, TNF-α, and IL-8 are secreted by the FM and promote the production of prostaglandins and proteases in the gestational compartment (Norwitz et al., 1992; Mitchell et al., 1993; Brown et al., 1998; Young et al., 2002; Myatt and Sun, 2010). Prostaglandins play a direct role in stimulating uterine contractions and cervical ripening, and proteases and ROS contribute to the weakening of the FM (Woods, 2001; Romero et al., 2014). The p38 MAPK pathway is critical for the initiation of cellular senescence and FM weakening, ultimately leading to membrane rupture (Menon et al., 2014). Increased generation of ROS in the gestational compartment is thought to activate the p38 pathway, leading to membrane senescence, damage to collagen, and weakening of the membranes in preparation for rupture in both term labor and pPROM (Woods, 2001).

Toxicants such as cigarette smoke extract and PBDEs activate one or more of these pathways in *in vitro* models of FM tissue or cells. For example, PBDEs induced oxidative stress, p38 MAPK activation and increased expression of cyclooxygenase-2 (a rate limiting enzyme of prostaglandin production) in human amnion epithelial cells (Behnia et al., 2015). Similarly, Menon et al. (2014) showed that cigarette smoke extract induced oxidative stress (assessed via formation of 3-nitrotyrosine staining) and activated the p38 MAPK pathway in FM explants *in vitro*. In addition, the environmental contaminant 2,3,7,8-tetrachlorodibenzo-p-dioxin (TCDD), often referred to as dioxin, increased expression of protease genes in human amnion epithelial cells (Abe et al., 2006) and increased a marker of senescence (β-galactosidase) in a FM “organ-on-chip” system consisting of primary human amnion epithelial cells co-cultured with decidual cells (Richardson et al., 2019). Thus, toxicology studies support molecular mechanisms that may explain epidemiological associations between toxicant exposures and adverse pregnancy outcomes mediated by the FM. However, several aspects of these phenomena, such as the thresholds of exposure and potential dimorphic responses based on fetal sex, remain largely unexplored.

## Toxicant-Pathogen Interactions: Impact on Fetal Membranes

Whereas several mechanisms have been identified which present plausible explanations for FM toxicity, immunomodulation in conjunction with bacterial infection remains an important but understudied phenomenon in gestational tissues. In a 2010 review, Feingold et al. (2010) highlighted the need for environmental toxicology research to incorporate interactions with infectious pathogens such as bacteria and viruses. Feingold et al. (2010) described four potential toxicant-pathogen interactions that could lead to disease: (1) toxicant and pathogen are both needed to cause disease; (2) pathogen and toxicant are individually capable of causing disease; (3) the chemical toxicant modifies the pathogen which leads to disease; and (4) the pathogen modifies the toxicant which leads to disease. In the same journal issue, Birnbaum and Jung (2010) called for increased attention to environmental health and infectious disease, noting that they can act concurrently, antagonistically, or synergistically. Despite this call to action, little research on toxicant-pathogen interactions during pregnancy has been conducted in the last decade.

### Toxicant-Pathogen Co-treatment Leads to Enhanced Inflammation

Some toxicants have been shown to enhance pathogen-stimulated oxidative stress pathways and pro-inflammatory responses in gestational tissues. For example, some PBDEs increased *E. coli*-stimulated IL-1β and IL-6 secretion and COX-2 expression, as well as reduced *E. coli*-stimulated IL-10 release in human placental explants (Peltier et al., 2012; Arita et al., 2018c). Similarly, TCDD increased bacteria-stimulated PGE_2_ and COX-2 gene expression and decreased IL-10 secretion (Peltier et al., 2013). Notably, the PBDE and TCDD effects were observed in the absence of impacts on explant viability and in placenta tissue obtained from both term (Arita et al., 2018c) and preterm (Peltier et al., 2012, 2013) stages of pregnancy, suggesting that immunomodulatory effects can occur throughout gestation. In addition, tributyltin enhanced *E. coli*-stimulated IL-6 release from placental explants (Arita et al., 2018a). Another study found that the flame retardant chemical tetrabromobisphenol A (TBBPA) increased the *E. coli*-induced release of IL-6 and TNF-α (Arita et al., 2018b). Research continues to be limited on how these toxicants modify bacterial host response in the FM and *in vivo*. Given the important nature of the FM in pPROM and PTB it is imperative that more research be conducted on toxicant-pathogen interactions in the FM.

### Immunosupression as a Mechanism of Toxicity

Whereas some toxicants enhance inflammation and immune responses, others have demonstrated immunosuppressive effects (Selgrade, 2007). Examples of toxic substances that suppress immune responses include alcohol, cigarette smoke, and air pollution, all of which have been shown to inhibit macrophage phagocytosis (Karavitis and Kovacs, 2011). Epidemiology studies have found associations between decreased antibody responses to vaccinations in children exposed to perfluorinated compounds (Grandjean et al., 2012) and polychlorinated biphenyls (Heilmann et al., 2006). Immunosuppressive effects of toxicants have also been observed in gestational tissues. For example, TBBPA and tributyltin both inhibited bacteria-stimulated IL-1β secretion in placental explants (Arita et al., 2018a, b).

### Immunomodulatory Effects of Trichloroethylene

The common environmental contaminant TCE is a well-documented example of a compound with immunosuppressive effects. Trichloroethylene is a chlorinated volatile organic solvent commonly used as an industrial metal degreaser (Waters et al., 1977; Chiu et al., 2013). Trichloroethylene is ranked #16 on the U.S. Agency for Toxic Substances and Disease Registry’s Priority List of Hazardous Substances and is a common environmental contaminant found in approximately 800 Environmental Protection Agency-designated Superfund sites (Wong, 2004; Chiu et al., 2013). Trichloroethylene is classified as a “known human carcinogen” (Guha et al., 2012) and is a renal and hepatic toxicant However, effects of TCE on gestational tissues have been minimally explored. Because of its continued industrial use and widespread persistent environmental contamination, TCE exposure continues to pose a threat to human health through ingestion of contaminated drinking water and inhalation of the volatilized chemical (Watson et al., 2006; Dumas et al., 2018). Trichloroethylene and its metabolites are detected in the blood of pregnant women exposed via inhalation and transfer across the placenta has been indicated by detection in the umbilical vein and artery (Beppu, 1968; Laham, 1970). Trichloroethylene and its metabolites are also found in the placenta and amniotic fluid of exposed pregnant mice (Ghantous et al., 1986). Thus, the effect of TCE and its downstream metabolites on gestational tissues in exposed women is of relevant concern.

The immunomodulatory effects of TCE are well documented in rodent and epidemiology studies. Mice co-treated with TCE and *Streptococcus zooepidemicus* showed increased mortality, decreased bacterial clearance from the lungs, and decreased alveolar phagocytosis (Aranyi et al., 1986; Selgrade and Gilmour, 2010). Trichloroethylene also suppressed activity of natural killer cells isolated from exposed rats (Wright et al., 1991). Immunosuppressive effects are observed in humans exposed to TCE. For example, lymphocyte counts as well as circulating levels of proinflammatory cytokines IL-6 and TNF-α were lower in exposed workers compared to controls (Hosgood et al., 2011; Xueqin et al., 2018). In a 2009 review, Cooper et al. (2009) concluded that “studies in mice and humans support an etiologic role of TCE in autoimmune disease.” It appears that metabolism is required for at least some of TCE’s immunotoxicity, because inhibition of the TCE metabolizing enzyme CYP2E1 mitigates some of these effects (Griffin et al., 2000). Despite these compelling findings, few researchers have investigated this phenomenon in gestational tissues such as the FM. Because the FM play a vital role in protecting the fetus and gestational compartment from pathogenic infection during pregnancy, an increased understanding of how environmental contaminant exposures modify FM responses to infection could greatly improve our ability to identify populations at risk for bacterial infection and associated adverse pregnancy outcomes.

### TCE Metabolite Suppression of Immune Responses to Bacteria in Fetal Membranes

Work in our laboratory demonstrated that the bioactive TCE metabolite S-(1,2-dichlorovinyl)-L-cysteine (DCVC) inhibits innate immune responses to GBS. These findings were observed in FM tissue explants (tissue cultures established from FM obtained from planned caesarian deliveries). Explants co-treated with GBS and DCVC showed decreased expression of TNF-α, IL-1β, and IL-8 compared to those treated with GBS alone (Boldenow et al., 2015). Two other TCE metabolites (TCA and DCA) showed no effect (Boldenow et al., 2015). Importantly, the concentrations of DCVC used (5–10 μM) were within the range of metabolite blood concentrations in female volunteers exposed to airborne TCE at the current occupational exposure limit (Lash et al., 1999; Agency for Toxic Substances and Disease Registry, 2007). Moreover, the immunomodulatory effects of DCVC occurred in the absence of any effect on overall GBS viability. The cytokine suppression occurred not only in response to GBS, but also in response to lipoteichoic acid and lipopolysaccharide (virulence factors expressed by multiple species of bacteria) (Alexander and Rietschel, 2001; Ginsburg, 2002), suggesting that the observed effects were not pathogen specific.

Suppression of cytokine expression has important implications for innate immune responses in FM. Cytokines play important roles during bacterial infection, such as the recruitment of immune cells. Thus, suppression of these cytokine responses could lead to decreased recruitment of immune cells during bacterial infection, leading to prolonged or more severe pathogenic infections during pregnancy. Prolonged or more severe infections could in turn lead to pPROM, PTB or other adverse pregnancy outcomes such as neonatal sepsis.

**FIGURE 1:**
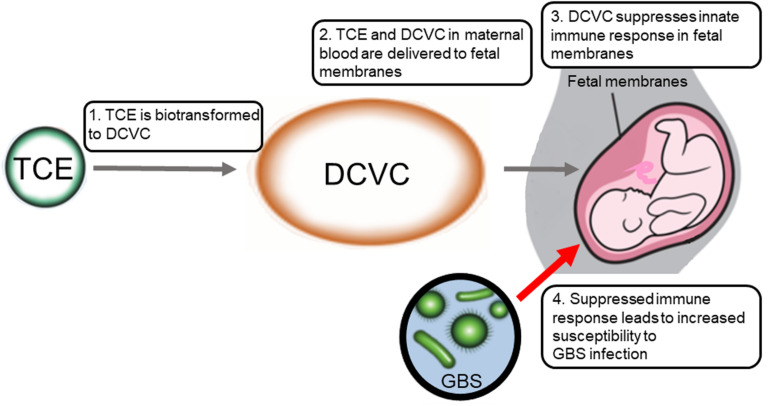
Proposed model of TCE immunosuppression in fetal membranes leading to increased susceptibility to GBS infection during pregnancy.

Figure 1 summarizes the major events in the proposed mechanism by which TCE exposure could lead to increased susceptibility to GBS infection. Few epidemiology studies have assessed associations between TCE exposure and PTB. Studies thus far have found associations with small for gestational age, low birth weight and birth defects but not PTB (Bove et al., 2002; Forand et al., 2012; Ruckart et al., 2014). However, these studies did not report on presence or absence of maternal pathogenic infection as a variable and obtaining accurate assessments of TCE exposure is challenging (Bove et al., 2002). Future epidemiology studies focusing on potential toxicant-pathogen interactions could greatly improve our understanding of whether phenomena observed in FM models *in vitro* translate to *in vivo* human outcomes.

## Discussion

Despite intriguing findings, numerous aspects of toxicant-pathogen interactions in FM need to be clarified in order to reach conclusions about the implications for maternal or fetal health outcomes. Although DCVC suppression of innate immune responses could exacerbate GBS infection, proinflammatory pathways are also critical in the activation of parturition, meaning that DCVC could also suppress activation of labor processes (Figure 1). A better understanding of mechanisms underlying these phenomena would clarify the true level of risk for adverse pregnancy outcomes due to GBS infection combined with TCE exposure. In addition, findings thus far have only been observed in FM tissue *in vitro*. While useful, these models lack a number of tissue interactions between the decidua and the chorionic layer of the FM as well as maternal immune responses to infection. Validating these findings in pregnant animal models co-treated with GBS and TCE could provide important clarification in this area. Furthermore, clarification is needed on whether DCVC is the sole metabolite of TCE responsible for immunosuppressive effects or if downstream metabolites play a role. Improved understanding of virulence factors that allow bacteria such as GBS to evade the defenses of the FM and colonize the amniotic fluid and/or fetus would also represent a significant step forward. Fetal sex and gestational age are other potentially important variables that were not considered in prior studies of the FM. Finally, TCE is far from the only toxicant known to have immunosuppressive effects. For example, perfluorinated chemicals such as perfluorooctanoic acid have recently generated concern due to observed immunosuppressive effects (Shane et al., 2020) and therefore should be investigated for interactions with pathogens in the context of pregnancy. Other classes of chemicals that have demonstrated immunosuppressive effects include aromatic hydrocarbons, benzene and metals such as lead and arsenic (National Academy of Sciences, 1992).

Some studies have noted both immunosuppressive and immune activation effects for the same toxicant. For example, Arita, et al. observed an increase in E. coli-induced TNF-α in placental explants treated with TBBPA, whereas IL-1β secretion was reduced (Arita et al., 2018b). This is not surprising given the inherent complexity of immunological signaling pathways. For a given toxicant, it is possible that both immune activation and suppression could occur to differing degrees simultaneously or in sequence, which is especially important to recognize when utilizing *in vitro* models. For example, if cultured FM are exposed to the toxicant and pathogen simultaneously then the toxicant may not diffuse into the tissue before the pathogen stimulates the early TNF-α response, but the toxicant could still inhibit the later IL-1β response. Additionally, the toxicant may act to inhibit or activate different molecular pathways within the immune system. For example, the toxicant could be inhibiting caspase, which is needed for IL-1β secretion, while simultaneously activating TNF-α (Thornberry et al., 1992). If immune activation predominates, adverse pregnancy events may include preterm activation of labor pathways which could lead to premature rupture of the FM. If immune suppression is the dominant process, adverse events could include increased pathogenic infiltration into the gestational compartment due to inadequate FM immune response. The mechanisms determining whether suppression or activation predominate in the FM in response to toxicants are not currently well understood but are likely mediated by a number of factors including the dose of toxicant, stage of pregnancy, strain or species of pathogen or duration of toxicant exposure (e.g., chronic vs. acute exposure). For example, naturally occurring immunological changes occurring throughout pregnancy include a progressive increase in the number and responsiveness of circulating neutrophils (Aghaeepour et al., 2017). Therefore, being exposed to a toxicant and/or pathogen late in pregnancy may favor immune activation whereas a different response may be observed with exposure earlier in pregnancy. Whether toxicant immune activation, suppression or a more complex interaction between the two, is the most relevant to the FM for a given toxicant-pathogen interaction is difficult to predict, further highlighting the need for additional research on this topic.

**FIGURE 2:**
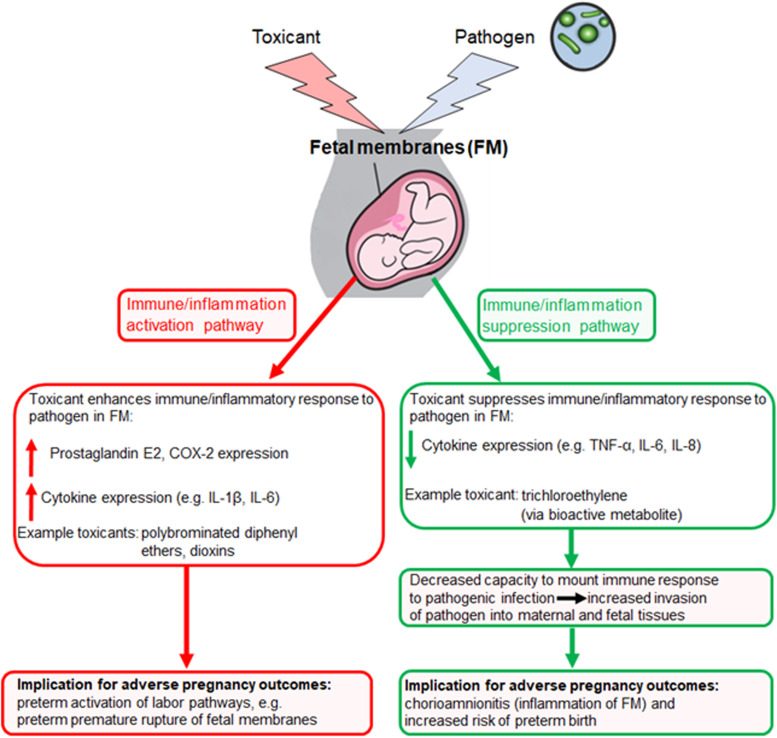
Proposed pathways for toxicant activation or suppression of inflammation/immune responses in the fetal membranes with potential implications for pregnancy outcomes. Multiple environmental toxicants have been identified that either enhance or suppress immune responses in the fetal membranes, particularly in models of pathogenic infection. Both mechanisms of toxicity have potentially significant implications for adverse pregnancy outcomes, e.g., early activation of labor pathways (activation) or decreased capacity for membrane tissue to mount a defense against pathogens (suppression). These pathways may not be mutually exclusive.

In summary, limited studies have shown that toxicants can potentially modify immune responses in the FM through both “immune/inflammation activation” and “immune/inflammation suppression” pathways (see Figure 2). Because current research into these phenomena has relied mostly on *in vitro* models of gestational cells and tissues, further research is needed to determine whether effects observed *in vitro* are replicated in FM *in vivo*. *In vitro* models are necessarily removed from the inherent complexity of the *in vivo* immune system. Studies using animal models would improve our understanding of how toxicants affect immune responses in the FM in an intact organism. Further research could improve our understanding of toxicant-pathogen interactions during pregnancy and potentially identify populations at risk for adverse pregnancy outcomes.

## Data Availability Statement

The raw data supporting the conclusions of this article will be made available by the authors, without undue reservation, to any qualified researcher.

## Author Contributions

EB, SH, and RL-C proposed the original idea for the manuscript. All authors wrote and edited the manuscript and have seen and approved the final version of the submitted manuscript.

## Conflict of Interest

The authors declare that the research was conducted in the absence of any commercial or financial relationships that could be construed as a potential conflict of interest.
